# Refeeding activates neurons in the dorsomedial hypothalamus to inhibit food intake and promote positive valence

**DOI:** 10.1016/j.molmet.2021.101366

**Published:** 2021-10-30

**Authors:** Daigo Imoto, Izumi Yamamoto, Hirokazu Matsunaga, Toya Yonekura, Ming-Liang Lee, Kan X. Kato, Takeshi Yamasaki, Shucheng Xu, Taiga Ishimoto, Satoshi Yamagata, Ken-ichi Otsuguro, Motohiro Horiuchi, Norifumi Iijima, Kazuhiro Kimura, Chitoku Toda

**Affiliations:** 1 Laboratory of Biochemistry, Graduate School of Veterinary Medicine, Hokkaido University, Sapporo, Hokkaido, 060-0818, Japan; 2 Laboratory of Animal Experiment, Institute for Genetic Medicine, Hokkaido University, Sapporo, 060-0815, Japan; 3 Laboratory of Pharmacology, Graduate School of Veterinary Medicine, Hokkaido University, Sapporo, 060-0818, Japan; 4 Laboratory of Veterinary Hygiene, Graduate School of Veterinary Medicine, Hokkaido University, Sapporo, 060-0818, Japan; 5 National Institutes of Biomedical Innovation, Health and Nutrition, Ibaraki, Osaka, 567-0085, Japan; 6 Immunology Frontier Research Center, Osaka University, Suita, Osaka, 565-0871, Japan

**Keywords:** Refeeding, Hypothalamus, Satiety, Opioid, Positive valence

## Abstract

**Objective:**

The regulation of food intake is a major research area in the study of obesity, which plays a key role in the development of metabolic syndrome. Gene targeting studies have clarified the roles of hypothalamic neurons in feeding behavior, but the deletion of a gene has a long-term effect on neurophysiology. Our understanding of short-term changes such as appetite under physiological conditions is therefore still limited.

**Methods:**

Targeted recombination in active populations (TRAP) is a newly developed method for labeling active neurons by using tamoxifen-inducible Cre recombination controlled by the promoter of activity-regulated cytoskeleton-associated protein (Arc/Arg3.1), a member of immediate early genes. Transgenic mice for TRAP were fasted overnight, re-fed with normal diet, and injected with 4-hydroxytamoxifen 1 h after the refeeding to label the active neurons. The role of labeled neurons was examined by expressing excitatory or inhibitory designer receptors exclusively activated by designer drugs (DREADDs). The labeled neurons were extracted and RNA sequencing was performed to identify genes that are specifically expressed in these neurons.

**Results:**

Fasting-refeeding activated and labeled neurons in the compact part of the dorsomedial hypothalamus (DMH) that project to the paraventricular hypothalamic nucleus. Chemogenetic activation of the labeled DMH neurons decreased food intake and developed place preference, an indicator of positive valence. Chemogenetic activation or inhibition of these neurons had no influence on the whole-body glucose metabolism. The labeled DMH neurons expressed prodynorphin (pdyn), gastrin-releasing peptide (GRP), cholecystokinin (CCK), and thyrotropin-releasing hormone receptor (Trhr) genes.

**Conclusions:**

We identified a novel cell type of DMH neurons that can inhibit food intake and promote feeding-induced positive valence. Our study provides insight into the role of DMH and its molecular mechanism in the regulation of appetite and emotion.

## Highlights

• Fasting-refeeding activates a subset of neurons in the dorsomedial hypothalamus (DMH).
• Chemogenetic inhibition of the DMH neurons increases food intake.
• Chemogenetic activation of the DMH neurons inhibits food intake and promotes positive valence.
• The DMH neurons express pdyn, GRP, CCK and Trhr genes.

## Graphical abstract

**Figure:**
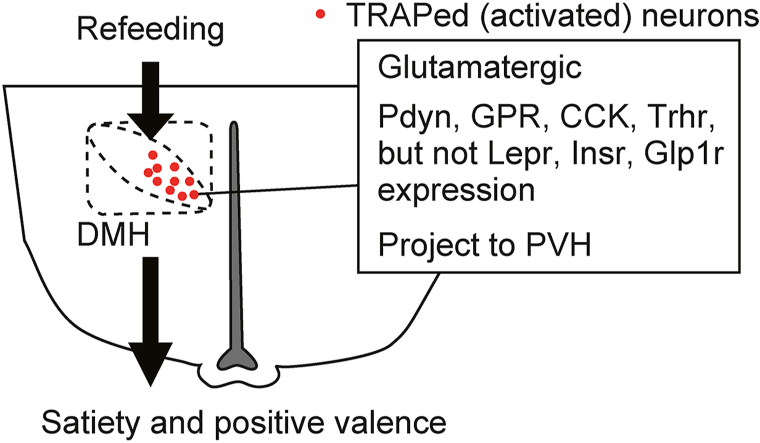

## 1 Introduction

An imbalance between food intake and energy expenditure leads to various health problems, including obesity. Food intake is regulated by homeostatic controls of appetite that induce hunger (feeding), satiation (suppression of feeding), and satiety (post-meal termination of hunger) [1,2]. Recent studies have identified neurons and neuronal circuits that regulate food intake [[3], [4], [5], [6]]. For example, the arcuate nucleus of the hypothalamus (ARC) contains various cell types and receives endocrine and exogenous signals that regulate feeding behaviors. Ghrelin secreted from the stomach activates neurons that release neuropeptide Y (NPY) and agouti-related protein (AgRP) in the ARC, resulting in increased food intake [7]. In contrast, the activation of proopiomelanocortin (POMC)-releasing neurons and vesicular glutamate transporter 2 (VGLUT2)-expressing neurons in the ARC decreases food intake [8,9]. The paraventricular nucleus of the hypothalamus (PVH) contains two subnuclei—the ventral parvocellular and dorsal magnocellular regions—that have distinct neuronal projections. A subset of PVH neurons, expressing pituitary adenylate cyclase-activating polypeptide (PACAP) and thyrotropin-releasing hormone (TRH), activates NPY/AgRP neurons via glutamatergic neurotransmission [10]. NPY/AgRP neurons suppress other types of PVH neurons to promote satiety [10]. Although the dorsomedial hypothalamus (DMH) is less well studied than the ARC and PVH, NPY neurons and cholinergic neurons in this region are involved in the regulation of food intake [11,12].

Most studies on the neural regulation of feeding have used cell type-specific knockout performed with the Cre recombinase (Cre)/loxP system. While it is a powerful method for investigating the role of specific neurons and neuropeptides, it has methodological limitations. For example, AgRP knockout mice do not change feeding behavior even though the role of AgRP in appetite is critical after birth [13,14]. The permanent deletion of a gene has a long-term effect on neurophysiology, making it unsuitable for investigating short-term changes such as appetite. Gene deletions during the developmental stage may affect neuronal projections and brain structures. The role of these neuronal circuits in the regulation of food intake under normal physiological conditions remains unclear.

Recently, Guenthner et al. developed a new approach for labeling active neurons *in vivo*, called the targeted recombination in active populations (TRAP) method [15]. In this method, the promoter of the cFos or activity-regulated cytoskeleton-associated protein (Arc/Arg3.1) gene, which are members of the immediate early gene family that are employed as markers of neuronal activity, is used to express tamoxifen-dependent Cre. Tamoxifen injection induces the expression of a fluorescent protein in active neurons expressing cFos or Arc/Arg3.1. We selected Arc/Arg3.1 because it is a neuron-specific gene, while cFos is not [[15], [16], [17]].

In this study, we investigated the role of active hypothalamic neurons during the refeeding period using the TRAP method [15]. We observed an increase in TRAP-labeled neurons in the DMH, 1 h after refeeding. The chemogenetic activation of DMH neurons by excitatory designer receptors exclusively activated by designer drugs (DREADDs) decreased food intake, suggesting that these neurons are involved in the regulation of feeding behavior in mice. In addition, the activation of these neurons produced conditioned place preference (CPP), suggesting that they are involved in producing the reward effect (positive valence) during feeding. The RNA sequencing study revealed that the labeled neurons in the DMH expressed an opioid polypeptide, prodynorphin (pdyn) as well as cholecystokinin (CCK). Taken together, our findings reveal a novel type of neuron in the DMH that regulates the appetite satiety process and promotes positive valence in mice. This neuronal circuit could be a potential therapeutic target for obesity and eating disorders.

## 2 Material and methods

### 2.1 Animal ethics and husbandry

All animal experiments were approved by the Animal Care and Use Committee of Hokkaido University and were performed according to the institutional guidelines. Arg3.1-Cre/ER^T2^ and Ai14 mice were purchased from The Jackson Laboratory (Bar Harbor, ME, USA). Arg3.1-Cre/ER^T2^ mice were crossed with Ai14 mice to obtain Arg3.1-Cre/ER^T2^;Ai14 mice. In this study, Arg3.1-Cre/ER^T2^ and Arg3.1-Cre/ER^T2^;Ai14 mice (8–20 weeks of age) were used. All mice were kept at 22 ± 4 °C under a 12/12-h light/dark cycle (light phase: 7:00–19:00) and given *ad libitum* food and water access. Mice were fed normal diet (ND) from Nosan Corporation (Yokohama, Japan).

### 2.2 Targeted recombination in active populations (TRAP)

Male Arg3.1-Cre/ER^T2^;Ai14 mice were fasted overnight and fed ND *ad libitum* in the group-housed cage. The mice received 4-hydroxy tamoxifen (4-OHT) (10 mg/kg; Cat# H6278, Sigma–Aldrich, St. Louis, MO, USA) intraperitoneal (i.p.) injection at 0, 0.5, 1, or 2 h after refeeding to label neurons expressing the Arc/Arg3.1 gene.

### 2.3 Brain sectioning and immunohistochemistry

Mice were sacrificed using CO_2_ asphyxiation and perfused with heparinized saline. Brain samples were harvested and incubated in 4% paraformaldehyde (PFA) overnight. Brain sections (50 μm) were collected. Floating sections were incubated with rabbit anti-cFos antibody (1:200; Cat# sc-52; Santa Cruz Biotechnology, Denton, TX, USA), rabbit anti-GFP antibody (1:1,000; Cat# GFP-Rb-Af2020; Frontier Institute, Ishikari, Japan), mouse anti-cFos antibody (1:1,000; Cat# sc-166,940, Santa Cruz Biotechnology), anti-Arc antibody (1:1000; Cat#156-004; Synaptic systems, Göttingen, Germany) or rabbit anti-dynA antibody (1:200; Cat# H-021-03; Phoenix Pharmaceuticals, Burlingame, CA, USA) in staining solution (0.1 M phosphate buffer [PB]) overnight at room temperature. After rinsing with PB, sections were incubated with Alexa 488 anti-rabbit (IgG) (1:250; Cat# A11034; Life Technologies, Carlsbad, CA, USA) or Alexa 594 anti-mouse (IgG) (1:250; Cat# 8890; Cell Signaling Technology, Danvers, MA, USA) for 2 h at room temperature. Brain sections stained with guinea pig anti-tdTomato antibody (1:1,000; Cat# tdTomato-GP-Af430; Frontier Institute) were incubated with biotin-labeled anti-guinea pig IgG (1:250; Cat# A18773; Invitrogen, Carlsbad, CA, USA) overnight. After rinsing with PB, sections were incubated with Alexa 594 streptavidin (1:2,000; Cat# SA-5488; Life Technologies, Eugene, OR, USA) for 1 h at room temperature. Sections were then rinsed with PB and mounted on glass slides with Vectashield mounting medium with DAPI (Cat# H1200, Vector Laboratories, Burlingame, CA, USA). Fluorescent signals for EGFP and mCherry in Figure 2C (EGFP: rabbit anti-GFP; mCherry: guinea pig anti-tdTomato) and 4B–G (EGFP: rabbit anti-GFP) were enhanced by immunohistochemistry for visualization. Quantification of fluorescent signals was carried out using ImageJ [18]. Image colors used in Figure 2C were adjusted; EGFP (green) to red and cFos (red) to green using ImageJ for comparison.

**Figure 1:**
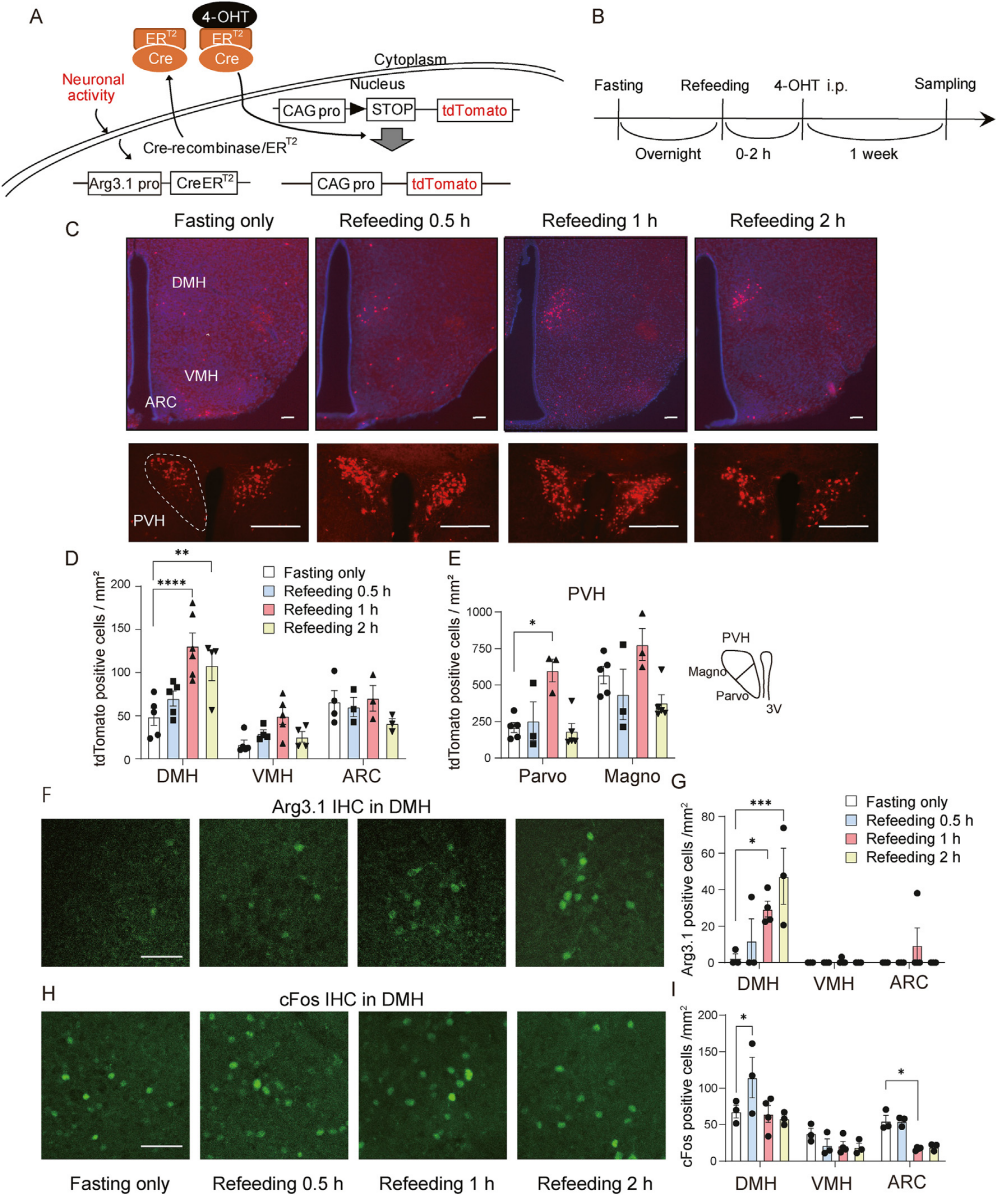
Fasting-refeeding activates neurons in the DMH. A Genetic strategy for visualizing activated neurons using the TRAP method. Neuronal activity promotes the expression of 4-hydroxy tamoxifen (4-OHT)-dependent Cre recombinase (Arg3.1-Cre/ER^T2^). The recombination of the reporter gene occurs in the presence of 4-OHT, allowing the expression of the tdTomato reporter gene for fluorescence visualization. B Experimental scheme. Male Arg3.1-Cre/ER^T2^; Ai14 mice were fasted overnight and re-fed normal diet (ND). 4-OHT intraperitoneal (i.p.) injection was given at 0, 0.5, 1, or 2 h after refeeding. Samples were collected 1 week after the injection. C Representative fluorescence images of TRAP-labeled tdTomato-positive neurons in the DMH, VMH, ARC and PVH at 0, 0.5, 1 or 2 h after refeeding. Sections were stained with DAPI (blue). Scale bar: 300 μm. D Quantification of TRAP-labeled neurons in the DMH (n = 4–6), VMH (n = 4 or 5) and ARC (n = 3) at 0, 0.5, 1 or 2 h after refeeding. E Quantification of TRAP-labeled neurons in the parvocellular and magnocellular regions of the PVH (n = 3–5). Schematic showing the parvocellular and magnocellular regions of the PVH. F Representative fluorescence images of Arg3.1 immunohistochemistry showing positive cells in the DMH at 0, 0.5, 1, or 2 h after refeeding. Scale bar: 50 μm. G Quantification of Arg3.1 positive cells in the DMH, VMH and ARC at 0 (n = 3), 0.5 (n = 3), 1 (n = 4) or 2 (n = 3) h after refeeding. H Representative fluorescence images of cFos immunohistochemistry showing positive cells in the DMH at 0, 0.5, 1, or 2 h after refeeding. Scale bar: 50 μm. I Quantification of cFos positive cells in the DMH, VMH and ARC at 0 (n = 3), 0.5 (n = 3), 1 (n = 4), or 2 (n = 3) h after refeeding. All data are shown as mean ± SEM. Two-way ANOVA, Tukey post hoc test. ∗∗P < 0.01; ∗∗∗∗P < 0.0001.

**Figure 2:**
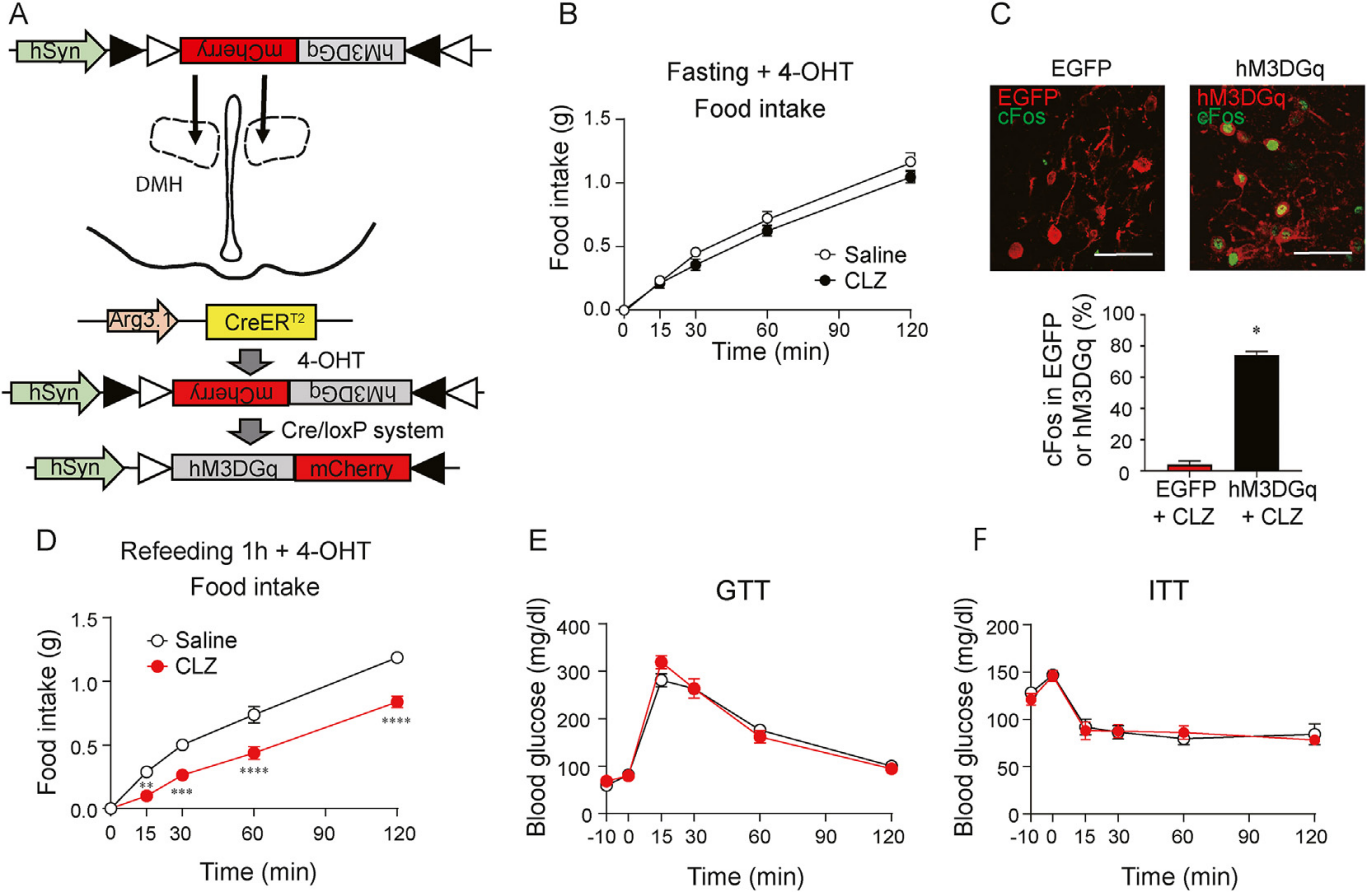
Chemogenetic activation of labeled DMH neurons decreases food intake without affecting glucose metabolism. A The AAV vector construct used for this experiment. Male Arg3.1-CreER^T^^2^ mice received bilateral AAV2-hSyn-DIO-hM3DGq-mCherry virus injection into the DMH. After recovery, the mice were fasted overnight and were given 4-OHT injection (i.p.) with or without refeeding to induce Cre recombination. B Food intake (0–120 min) after i.p. injection (−10 min) of CLZ (n = 9) or saline (n = 9) in the mice which were given 4-OHT injection (i.p.) without refeeding. C Representative images and quantification of the colocalization of cFos and EGFP (n = 3) or hM3DGq (n = 5). Mice were fasted overnight and were given 4-OHT at 1 h after refeeding. Scale bar: 50 μm. D Food intake (0–120 min) after i.p. injection (−10 min) of CLZ (n = 8) or saline (n = 8). Mice were fasted overnight and were given 4-OHT at 1 h after refeeding. E Glucose tolerance test (GTT) (0–120 min) after i.p. injection (−10 min) of CLZ (n = 8) or saline (n = 8). F Insulin tolerance test (ITT) (0–120 min) after i.p. injection (−10 min) of CLZ (n = 8) or saline (n = 8). Each point represents mean ± SEM. Two-way ANOVA, Sidak post hoc test. ∗P < 0.05; ∗∗P < 0.01; ∗∗∗P < 0.001; ∗∗∗∗P < 0.0001.

### 2.4 Stereotaxic surgeries and adeno-associated virus (AAV) injection

Arg3.1-Cre/ER^T2^ mice (12–20-week-old) were anesthetized with a mixture of ketamine (100 mg/kg) and xylazine (10 mg/kg), and positioned on a stereotaxic instrument (Narishige, Tokyo, Japan). Mice were injected in each side or one side of the DMH with ∼0.5 μL AAV2-hSyn-DIO-hM3D (Gq)-mCherry (Addgene, Cambridge, USA) [19], AAV2-hSyn-DIO-hM4D (Gi)-mCherry (Addgene) [19], AAV8-eSyn-DIO-hChR2(H134R)-EGFP (Vector Biolabs, Malvern, PA, USA) or AAV8-eSyn-DIO-eNpHR3.0-EYFP (Vector Biolabs) using the following coordinates: AP: −1.85 mm, L: ± 0.3 mm, DV: −5.5 mm. A stainless-wall guide and an internal cannula (P1 Technologies, Roanoke, VA, USA; guide cannula, C235G/SPC gauge 26; internal cannula, C2351/SPC gauge 33) were used for the injection of AAV. An injection with 0.2 μL of AAV solution did not achieve sufficient expression of mCherry or EGFP, probably due to a leakage of AAV in the space between guide and internal cannula. Thus, we used 0.5 μL of the AAV solution. Open wounds were sutured after viral injection. A 7- to 14-day recovery period was allowed before starting the experiments. Mice were fasted overnight and received 4-OHT (10 mg/kg) injection (i.p.) during fasting to induce Cre recombination. After finishing measurements of food intake, glucose metabolism and place preference behavior, the same mice received 4-OHT 1 h after refeeding. To ensure adequate expression of proteins, experiments were carried out at least 7 days after the 4-OHT injection.

### 2.5 Glucose and insulin tolerance test

Glucose and insulin tolerance tests were performed on fasted mice expressing DREADDs (hM3DGq or hM4DGi) to assess the role of refeeding-responsive DMH neurons. To activate DREADDs, clozapine (CLZ) solution (0.1 mg/kg; Cat# C6305, Sigma–Aldrich, St. Louis, MO, USA) was injected (i.p.) 10 min or 30 min before glucose or insulin tolerance tests. We chose CLZ instead of clozapine N-oxide (CNO), because CLZ can activate DREADD receptors at a lower concentration than CNO [20]. For the glucose tolerance test, the animals were fasted overnight and injected with glucose (2 g/kg; i.p.). Blood glucose levels were measured with a handheld glucose meter (Nipro Free Style, Nipro, Osaka, Japan) before injection of CLZ (−10 or −30 min) and glucose (0 min), as well as 15, 30, 60, and 120 min after glucose injection. The insulin tolerance test was performed on *ad libitum*-fed mice. The mice were injected with 0.75 U/kg insulin (Novo Nordisk, Bagsværd, Denmark). Blood glucose was measured before injection of CLZ (−10 or −30 min) and insulin (0 min) as well as at 15, 30, 60, and 120 min after insulin injection.

### 2.6 Food intake measurement

The animals were placed in a new cage (single cage housing) the day before the experiment. Food was removed before the beginning of the dark phase, and the mice were fasted overnight. The animals received saline or CLZ (0.1 mg/kg) injection (i.p.) 30 min before refeeding. Food intake was measured at 0, 15, 30, 60, and 120 min.

### 2.7 Conditioned place preference (CPP) test

The CPP apparatus consisted of compartments distinguished by a yellow striped floor (with stripes) and a white floor (no stripes) connected by an adjacent aisle. All experiments were carried out in the light phase. The protocol for the CPP test was as described by Kim et al. [21]. Test and conditioning times were measured with a stopwatch, and the video recording was analyzed using ImageJ [18] and MouBeAT [22]. On day 1, male Arg3.1-Cre/ER^T2^ mice expressing hM3DGq or EGFP were placed in the CPP apparatus and allowed to explore freely for 10 min without saline or CLZ injection to determine intrinsic place preference. The animals with intrinsic place preference index (PPI) > 50% were excluded from the analysis. PPI (%) was calculated using Equation (1)

(1) $$\text{PPI }\left(\text{\%}\right)=\frac{\text{ Time spent in the unpreferred chamber }\left(\text{no stripe}\right)-\text{Time spent in the preferred chamber }\left(\text{with stripe}\right)}{\text{Time spent in either chamber}}\times 100$$

Conditioning took place between days 2–5, where the animals had access to only one chamber. On days 2 and 4, the mice received CLZ injection (i.p.) 40 min prior to the experiment and were placed in a chamber without stripes for 30 min. On days 3 and 5, the mice received saline injection (i.p.) 40 min prior to the experiment and were placed in a chamber with stripes for 30 min. On day 6, the mice freely explored the entire CPP apparatus for 10 min without saline or CLZ injections, and the time spent in each chamber was video recorded. Normalized place preference was calculated using Equation (2).

(2) Normalized place preference(%)=PPI(post-test)−PPI(pre-test)

### 2.8 Cell sorting for RNA sequencing

TRAP-labeled refeeding-responsive DMH neurons were collected by sorting tdTomato-positive neurons from the DMH of male Arg3.1-Cre/ER^T2^;Ai14 mice. Male Arg3.1-Cre/ER^T2^;Ai14 mice (8- to 12-week-old) were fasted overnight and given 4-OHT injection 1 h after refeeding with ND the next day. Sample collection was carried out 1 week after the injection.

We used a modification of a protocol reported previously for manually sorting tdTomato-positive and negative neurons [23]. Mice were sacrificed using CO_2_ asphyxiation, and the brain was placed in ice-cold cutting solution (220 mM sucrose, 2.5 mM KCl, 6 mM MgCl_2_, 1 mM CaCl_2_, 1.25 mM NaH_2_PO_4_, 10 mM glucose, 26 mM NaHCO_3_, bubbled thoroughly with 95% O_2_/5% CO_2_). A coronal brain slice (500 μm) containing the DMH was obtained using a vibratome (PELCO easiSlicer, Redding, CA, USA). The DMH was dissected and placed in a tube containing filtered artificial cerebrospinal fluid (ACSF; 105 mM NaCl, 2.5 mM KCl, 2 mM CaCl_2_, 1.3 mM MgSO_4_, 1.23 mM NaH_2_PO_4_, 24 mM NaHCO_3_, 20 mM HEPES, 2.5 mM glucose, 100 nM TTX, 20 μM DQNX, 50 μM AP-V, pH 7.4, bubbled thoroughly with 95% O_2_/5% CO_2_) with papain (0.3 U/ml), DNase (0.075 μg/ml) and BSA (3.75 μg/ml). The tube was incubated on a shaker (34 °C, 75 rpm) for 15 min. After incubation, the tissue was washed three times with papain-free filtered ACSF supplemented with FBS (1%). Following this, 1 ml ACSF with FBS (1%) was added, and the samples were triturated successively with 600, 300, and 150 μm fire-polished Pasteur pipettes (10 times each). The tube was centrifuged (120×*g*, 5 min, room temperature), and the supernatant was removed. The cell pellet was resuspended in 10 ml filtered ACSF containing FBS (1%) and transferred to a 100 mm collagen type I-coated petri dish. The cells were allowed to settle onto the floor of the dish for 10 min, and tdTomato-positive and negative neurons were sorted separately under the fluorescence microscope using a cell aspirator attached to a micropipette (diameter: 30–50 μm). The neurons were transferred to a clean 35 mm collagen type I-coated petri dish containing filtered ACSF with FBS (1%). This sorting process was repeated once more, and the cells were transferred to a 35-mm collagen type I-coated petri dish containing filtered PBS (bubbled thoroughly with 95% O_2_/5% CO_2_). The sorted neurons were transferred to a PCR tube (five neurons per tube) containing 1 μL of 10 × reaction buffer (SMART-Seq v4 Ultra Low Input RNA Kit for Sequencing, Cat. No. 634888, Takara Bio, Kusatsu, Japan). Nuclease-free water was added to 11.5 μL, and the mixture was stored at −80 °C until library preparation.

### 2.9 RNA sequencing

RNA sequence library preparation, sequencing, and mapping of gene expression were performed by DNAFORM (Yokohama, Kanagawa, Japan). Double-stranded cDNA libraries (RNA-seq libraries) were prepared using SMART-Seq v4 Ultra Low Input RNA Kit for Sequencing (Cat. No. 634888, Takara Bio) according to the manufacturer's instructions. RNA-seq libraries were sequenced using paired-end reads (50-nt read 1 and 25-nt read 2) on a NextSeq 500 instrument (Illumina, San Diego, CA, USA). The obtained reads were mapped to the mouse GRCm38.p6 genome using STAR (version 2.7.3a) [24]. Reads on annotated genes were counted using featureCounts (version 1.6.4) [25]. FPKM values were calculated from mapped reads by normalizing to total counts and transcript. Prism 8 software (GraphPad) was used to generate heatmaps. Gene lists used for RNA sequencing analysis were obtained from previous reports [26,27].

### 2.10 Statistical analysis

All data are presented as mean ± SEM, and *n* represents the number of animals. Statistical differences were evaluated with Student's unpaired *t-*test (for two-group comparisons), one-way ANOVA, or two-way ANOVA. Tukey or Sidak post-hoc tests (for multiple comparisons) were performed using Prism 8 software (GraphPad). Values of *P* < 0.05 were considered significant.

## 3 Results

### 3.1 Fasting-refeeding activates a subset of neurons in the compact part of the DMH

To identify neurons activated by refeeding, the TRAP method was used to label Arg3.1-expressing neurons. This method requires two transgenes: one expresses Cre recombinase fused with estrogen receptor type 2 (Arg3.1-Cre/ER^T2^) from an activity-dependent Arc/Arg3.1 promoter, and the other allows the expression of a tdTomato reporter for fluorescence visualization (Ai14:loxP-stop-loxP-tdTomato). 4-OHT, an active form of tamoxifen, was injected to induce translocation of Cre into the nucleus to cause recombination of the reporter gene. Continuous expression of the fluorescent proteins is driven by the CAG promoter (Figure 1A).

Male Arg3.1-Cre/ER^T2^;Ai14 mice were fasted overnight and re-fed ND. 4-OHT was injected (i.p.) at 0, 0.5, 1, or 2 h after refeeding, and the brain samples were collected 1 week later (Figure 1B). In the fasting only condition (0 h), we observed tdTomato-positive neurons in the DMH (48.8 ± 10.2 cells/mm^2^), VMH (16.9 ± 4.9 cells/mm^2^) and ARC (66.0 ± 13.0 cells/mm^2^) (Figure 1C,D). There was no significant change in the number of tdTomato-positive DMH neurons when 4-OHT was injected 0.5 h after refeeding compared with the fasting only condition (0 h). However, the number of tdTomato-positive neurons in the DMH increased 2.88 and 2.22-fold when 4-OHT was injected 1 and 2 h after refeeding, respectively (Figure 1D). There was no significant change in tdTomato-positive neurons in the VMH or ARC (Figure 1D). The tdTomato-positive neurons in the PVH were localized to the dorsal region at 0 h but were also detected in the parvocellular and magnocellular regions at 1 h after refeeding (Figure 1C). The distribution of tdTomato-positive neurons in the PVH were assessed according to the subdivisions described in the previous report [28] (Figure 1E). In the fasting only state (0 h), the number of tdTomato-positive neurons in the parvocellular and magnocellular regions were 209.2 ± 34.7 and 569.2 ± 59.3 cells/mm^2^, respectively. The number of tdTomato-positive neurons was increased 2.87-fold in the parvocellular region 1 h after refeeding, while no significant change was observed in the magnocellular region (Figure 1F). To confirm that refeeding activates DMH neurons, immunohistochemistry (IHC) of Arg3.1 or cFos was performed (Figure 1F–I). In agreement with the result of TRAP, Arg3.1-positive cells were increased in the DMH, but not VMH or ARC, after the refeeding (Figure 1F,G). In contrast, an increase in cFos expression in the DMH was found only 0.5 h after refeeding (Figure 1H,I). cFos expression in the ARC was decreased by refeeding (Figure 1I), which may represent that NPY/AgRP neurons are active during fasting [14]. These results suggest that fasting–refeeding activates neurons in the DMH.

### 3.2 Chemogenetic activation of TRAP-labeled DMH neurons decreases food intake

To understand the roles of labeled DMH neurons responding to refeeding, we used the targeted expression of genetically modified DREADDs that are activated exclusively by clozapine (CLZ) [20]. Male Arg3.1-CreER^T2^ mice used in the excitatory DREADD study received bilateral Cre-inducible-G_q_-DREADD virus (AAV2-hSyn-DIO-hM3DGq-mCherry) or control virus (AAV2-hSyn-DIO-EGFP) injection into the DMH. After 2 weeks of recovery, the animals were fasted overnight and 4-OHT was injected (i.p.) without refeeding to express excitatory DREADD in non-refeeding responsive neurons and non-DMH neurons (Figure 2A). The Arg3.1-CreER^T2^ mice were fasted overnight, injected with saline or CLZ, and food intake was measured (Figure 2B). In this condition, there was no difference in food intake between saline- and CLZ-treated mice (Figure 2B). The same Arg3.1-CreER^T2^ mice were fasted overnight, re-fed ND, and received a 4-OHT injection (i.p.) 1 h after refeeding to express excitatory DREADD in the DMH.

Activation of neurons by CLZ injection was confirmed by IHC for cFos expression. The percentage of colocalization of EGFP and cFos-expressing neurons was 4.17 ± 2.3% in the control group. The colocalization of excitatory DREADD and cFos-expressing neurons was 74.0 ± 2.5%, indicating that the DMH neurons expressing the excitatory DREADD were activated by CLZ stimulation (Figure 2C).

The effect of DMH neuron activation on food intake was explored using CLZ stimulation. The Arg3.1-CreER^T2^ mice expressing the excitatory DREADD in the DMH were fasted overnight and treated with saline or CLZ 10 min before refeeding with ND. The food intake values for the saline-treated group at 1 and 2 h after refeeding were 0.74 ± 0.07 g and 1.19 ± 0.13 g, respectively. In the CLZ-treated group, the food intake decreased significantly after refeeding compared with the saline-treated group (Figure 2D). The effect of activated neurons on glucose metabolism was also evaluated using the glucose tolerance test (GTT) and the insulin tolerance test (ITT). The animals received CLZ or saline injection 10 min before the tests. The blood glucose levels among the groups remained similar during the GTT and ITT (Figure 2E,F), indicating that the activation of these neurons has no impact on whole-body glucose metabolism. Our results show that the activation of refeeding-responsive DMH neurons reduces food intake without affecting glucose metabolism in mice.

### 3.3 Chemogenetic inhibition of TRAP-labeled DMH neurons increases food intake

**Figure 3:**
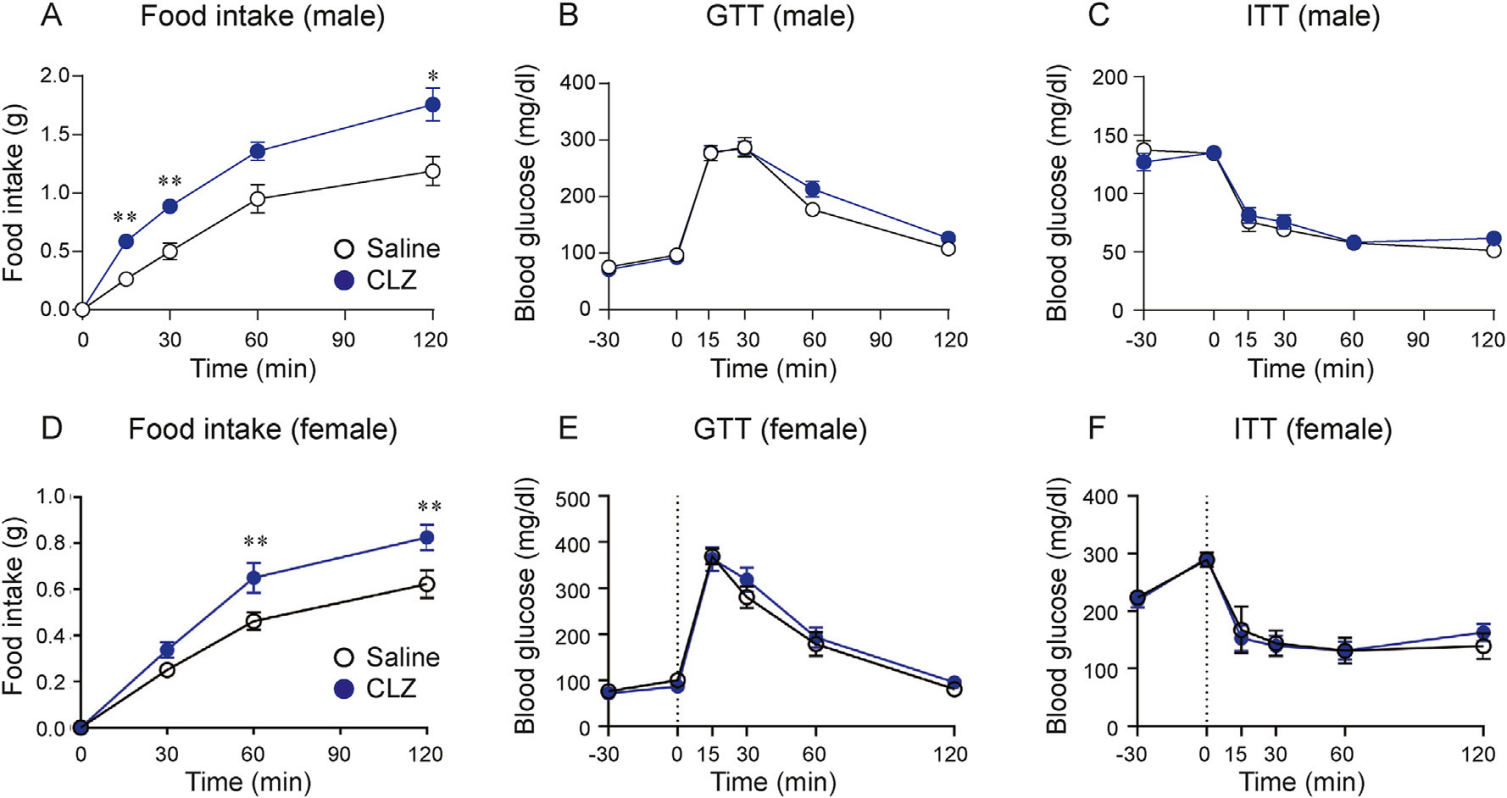
Chemogenetic inhibition of labeled DMH neurons increases food intake without impacting glucose metabolism. A Food intake (0–120 min) after i.p. injection (−30 min) of CLZ (n = 7) or saline (n = 8) in male Arg3.1-CreER^T^^2^ mice which received bilateral AAV2-hSyn-DIO-hM4DGi-mCherry virus injection into the DMH. 4-OHT was injected 1 h after refeeding. B, C GTT (B) and ITT (C) (0–120 min) after i.p. injection (−30 min) of CLZ (n = 8) or saline (n = 8) in male mice. D Food intake (0–120 min) after i.p. injection (−30 min) of CLZ (n = 12) or saline (n = 13) in female Arg3.1-CreER^T^^2^ mice which received bilateral AAV2-hSyn-DIO-hM4DGi-mCherry virus injection into the DMH. 4-OHT was injected 1 h after refeeding. E, F GTT (E) and ITT (F) (0–120 min) after i.p. injection (−30 min) of CLZ (n = 6) or saline (n = 6) in female mice. Each point represents mean ± SEM. Two-way ANOVA, Sidak post hoc test. ∗P < 0.05; ∗∗P < 0.01.

We further investigated the role of refeeding-responsive DMH neurons by using an inhibitory DREADD. Male Arg3.1-CreER^T2^ mice received bilateral injection of Cre-inducible-G_i_-DREADD virus (AAV2-hSyn-DIO-hM4DGi-mCherry) into the DMH. After a 2-week recovery, the mice were fasted overnight and received 4-OHT injection (i.p.) 1 h after refeeding. One week after the 4-OHT injection, food intake was measured. After overnight fasting, animals received either saline or CLZ injection (i.p.) 30 min before being re-fed ND. Food intake in the saline-treated mice after 0.5 and 2 h were 0.50 ± 0.07 g and 1.19 ± 0.12 g, respectively. The CLZ-treated mice significantly increased food intake (Figure 3A). Saline and CLZ-treated groups had similar blood glucose levels in GTT and ITT (Figure 3B,C). Female Arg3.1-CreER^T2^ mice expressing the inhibitory DREADD in the DMH were also used to measure food intake, GTT, and ITT (Figure 3D–F). The CLZ-treated mice significantly increased food intake but did not change blood glucose levels in GTT and ITT, suggesting that the inhibition of DMH neurons responding to refeeding increases food intake, which is not affected by sex differences.

### 3.4 Labeled DMH neurons project to the PVH, Shi, and LS

**Figure 4:**
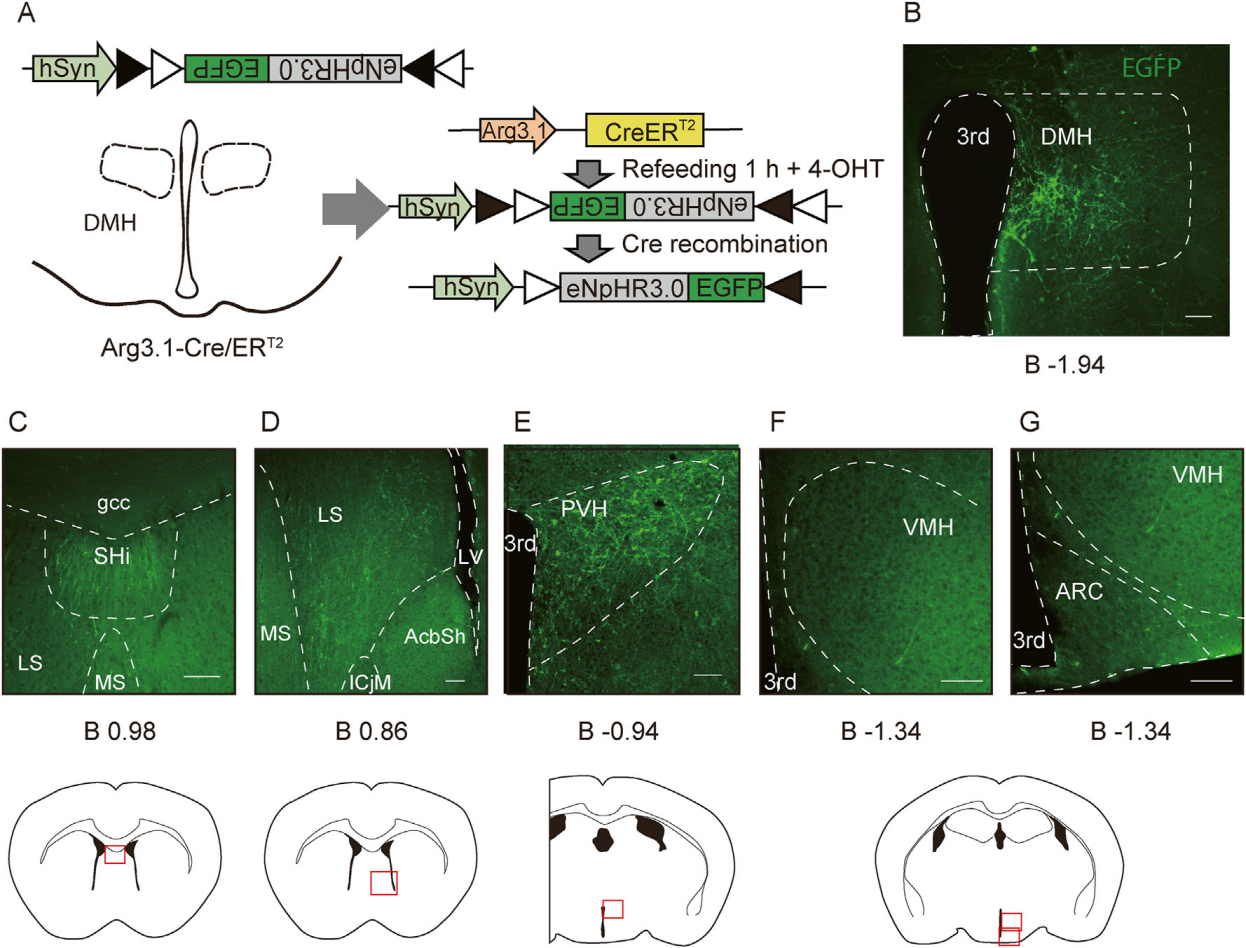
Neuronal projections of refeeding-responsive DMH neurons. A The AAV vector construct used for this experiment. Male Arg3.1-CreER^T^^2^ mice received AAV8-Syn-DIO-eNpHR3.0-EGFP virus injection into the DMH. After recovery, the mice were fasted overnight and re-fed ND. 1 h after refeeding, mice were given 4-OHT injection (i.p.) to induce Cre recombination (n = 3). B eNpHR3.0 (green) expression in the DMH (injection site). Scale bar: 100 μm. C-G eNpHR3.0 (green) expression in the SHi (C), LS (D), PVH (E) VMH (F) and ARC (G). Scale bar: 100 μm. SHi, septohippocampal nucleus; gcc, genu of the corpus callosum; LS, lateral septal nucleus; MS, medial septal nucleus; AcbSh, accumbens nucleus shell; ICjM, island of Calleja major island; LV, lateral ventricle; 3rd, third ventricle. Numbers under each brain slice show the distance from bregma (mm).

To identify the neural circuitry underlying the inhibition of food intake, we examined the projection site of the refeeding-responding DMH neurons using GFP-linked halorhodopsin (NpHR). NpHR is a light-driven chloride pump that is used as an inhibitory optogenetic actuator. NpHR is expressed in the cell body, as well as axons, dendrites, and their terminals, allowing identification of projection sites. Male Arg3.1-CreER^T2^ mice were injected Cre-inducible-NpHR virus (AAV8-Syn-DIO-eNpHR3.0-EGFP) into the DMH (Figure 4A). After a 2-week recovery period, the animals were fasted overnight and re-fed ND. One hour later, 4-OHT was injected (i.p.). Two weeks later, animals were perfused for analysis. GFP expression in the DMH was detected, indicating that refeeding-responding neurons were successfully infected by the NpHR virus (Figure 4B). Whole-brain regions were examined for GFP-positive fibers. We observed GFP expression in the septohippocampal nucleus (Shi), lateral septal nucleus (LS), and PVH, but not in other regions, including the ARC and VMH (Figure 4C–G). The DMH neuron may regulate food intake through PVH, SHi, or LS.

### 3.5 Chemogenetic activation of labeled DMH neurons induces conditioned place preference

**Figure 5:**
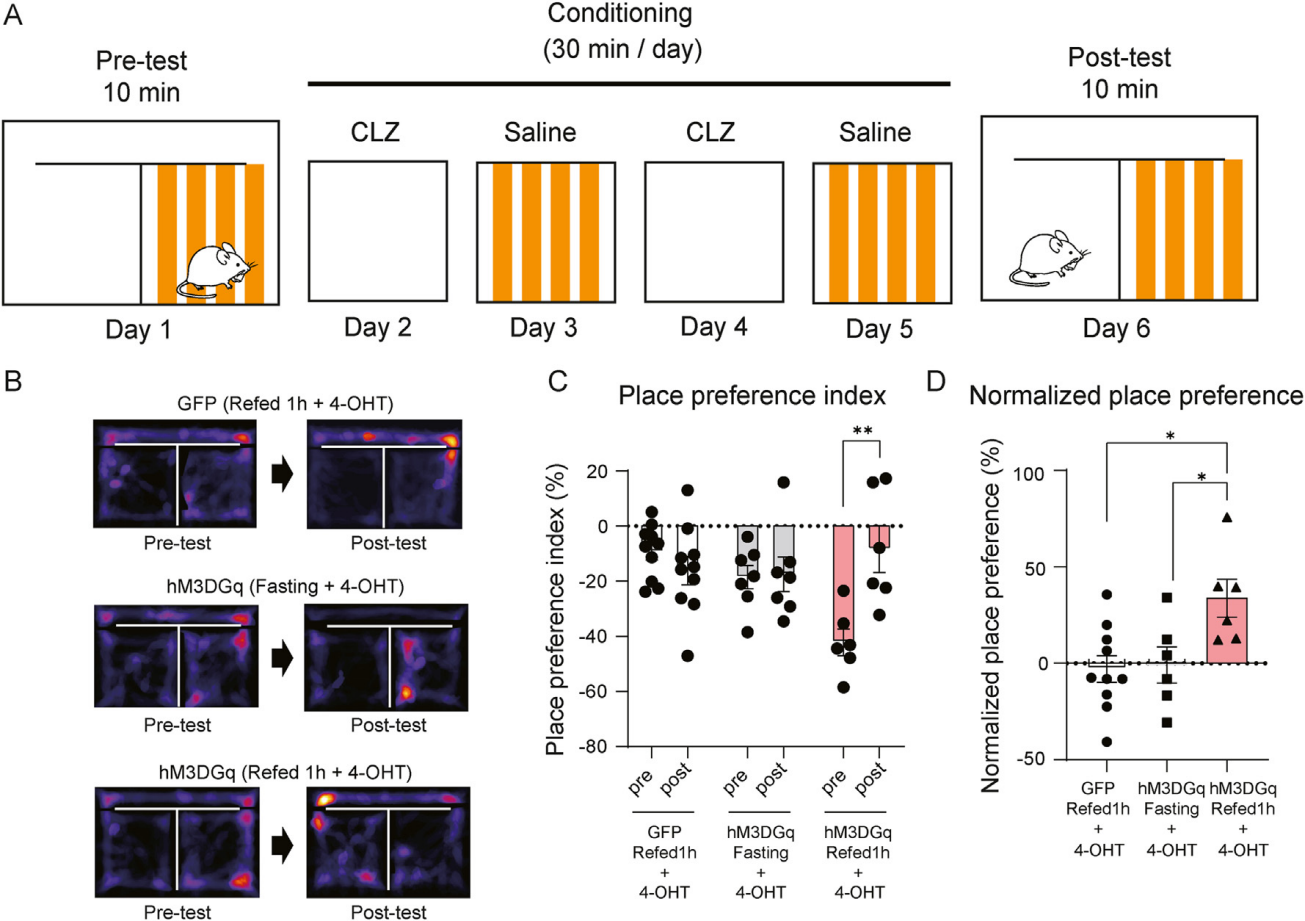
Intrinsic place preference is blocked by chemogenetic activation of labeled DMH neurons. A Scheme of the CPP test procedure: In the pre-test, excitatory DREADD (hM3DGq) or EGFP (control)-expressing male Arg3.1-CreER^T^^2^ mice were placed in the CPP apparatus for 10 min to determine intrinsic place preference (Day 1). The conditioning period was performed on alternate days (Days 2–5). The mice were placed on one side of the chamber for 30 min and received either CLZ (nonpreferred side) or saline (preferred side) injection (i.p.) on each day. In the post-test, the mice were placed in the CPP apparatus for 10 min for place preference assessment (Day 6). B Representative heatmaps showing the location of EGFP (control), hM3DGq (Fasting + 4-OHT) and hM3DGq (Refeeding1h + 4-OHT)-expressing mice in the pre- and post-tests. C Place preference index for EGFP (control) (n = 10), hM3DGq (Fasting + 4-OHT) (n = 7) and hM3DGq (Refeeding1h + 4-OHT) (n = 6)-expressing mice. Data are expressed as mean ± SEM. Two-way ANOVA, Sidak post hoc test. ∗∗P < 0.01. D Normalized place preference for EGFP (control) (n = 10), hM3DGq (Fasting + 4-OHT) (n = 7) and hM3DGq (Refeeding1h + 4-OHT) (n = 6)-expressing mice. Data are expressed as mean ± SEM. One-way ANOVA, Tukey post hoc test. ∗P < 0.05.

Our results suggest that the labeled DMH neurons participate in the regulation of food intake. Previous studies indicate that the brain circuits responsible for controlling appetite are also involved in regulating emotional processes, including positive emotion triggered by reward [[29], [30], [31]]. To investigate the role of labeled DMH neurons in emotion, we examined the effect of chemogenetic activation of these cells in the CPP test (Figure 5A). Male Arg3.1-CreER^T2^ mice expressing GFP (refeeding + 4-OHT), hM3DGq (fasting + 4-OHT) or hM3DGq (refeeding + 4-OHT) in DMH neurons were used for this experiment. The CPP apparatus contained two chambers distinguished by the patterns on the floor (with or without stripes). During the tests, the mice were allowed to move freely between the chambers (Figure 5A). On day 1 (pre-test), the mice were placed in the CPP apparatus for 10 min to determine intrinsic place preference. The time spent in each of the chambers was measured, and a heatmap was plotted. Before the conditioning phase, mice in all groups spent more time in the chamber with stripes (Figure 5B,C).

The conditioning was performed on alternate days. On days 2 and 4, mice received CLZ injections (i.p.) and were placed in the nonpreferred side (without stripes) for 30 min with a barrier in the hallway. On days 3 and 5, mice received saline injections (i.p.) and were placed in the preferred side (with stripes) for 30 min. On day 6 (post-test), the mice were placed in the CPP apparatus for 10 min without the barrier, and place preference was measured (Figure 5A). We observed a significant increase in the post-test PPI in the hM3DGq (refeeding 1h + 4-OHT) mice, but not in the GFP and hM3DGq (Fasting + 4-OHT) mice (Figure 5C; *P* < 0.01), indicating the hM3DGq (refeeding 1h + 4-OHT) group spent more time in the chamber without stripes. The normalized place preference also shows that the hM3DGq (refeeding 1h + 4-OHT) group spent more time in the chambers without stripes in the post-test compared with the GFP and hM3DGq (Fasting + 4-OHT) group (Figure 5D, *P* < 0.05). The conditioning phase had no impact on the preference in the control groups, indicating that the development of CPP was not affected by CLZ injection. Taken together, our results suggest that refeeding-responsive DMH neurons are involved in encoding the positive valence induced by refeeding.

### 3.6 Refeeding-responsive DMH neurons express Pdyn, GPR, CCK. and Trhr

The hypothalamus comprises various cell types, and their transcriptional profiles provide key information towards understanding this region's function in homeostatic regulation [26]. To investigate the transcriptional profile of tdTomato-positive DMH neurons, we performed an RNA-sequencing study using cells dissociated from male Arg3.1-Cre/ER^T2^;Ai14 mice.

**Figure 6:**
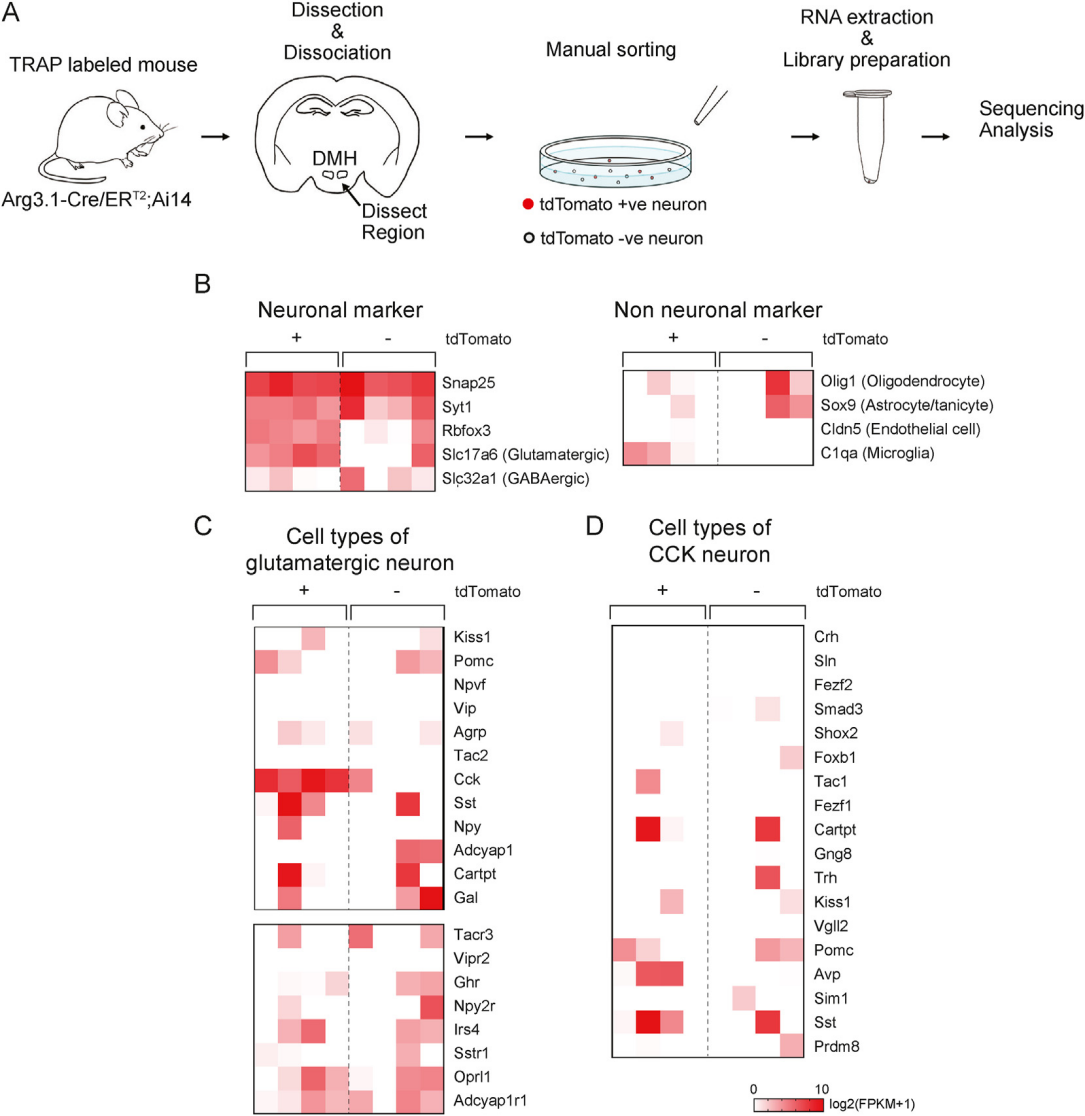
Refeeding-responsive DMH neurons express glutamatergic neuron markers. A Experimental scheme of the RNA sequencing protocol. Arg3.1-Cre/ER^T2^; Ai14 mice were TRAPed 1 h after refeeding. The DMH region was dissected and triturated for cell sorting. tdTomato-positive (+ve) and negative (−ve) neurons were collected manually for RNA sequencing. B Heatmaps representing the expression of neuronal and non-neuronal marker genes in tdTomato-positive and negative neurons. Log_2_ (FPKM + 1). C, D Heatmaps of the genetic markers for hypothalamic glutamatergic neurons. The gene list was obtained from a previous report [26]. Log_2_ (FPKM + 1).

After overnight fasting, mice were fed ND and given 4-OHT injection (i.p.) 1 h after refeeding. The tissue containing the DMH was dissected, and after cell dissociation, tdTomato-positive and negative (control) DMH neurons were sorted manually (5 cells per sample) for sequencing (Figure 6A). We generated gene expression heatmaps based on previous findings [26]. Pan neuronal markers, including Snap25 and Syt1, were detected in all samples (tdTomato-positive, *n* = 4; control, *n* = 4) (Figure 6B), suggesting successful sorting of tdTomato-negative (control) neurons, thereby permitting assessment of differential transcripts. As the probability of tdTomato-negative neurons being other non-neuronal cell types is high, relatively low expression of non-neuronal marker genes was observed in the control samples (Figure 6B). The expression of the glutamatergic neuron marker Slc17a6 was higher than that of the GABAergic neuron marker Slc32a1 in tdTomato-positive neurons (Figure 6B), suggesting that the refeeding-responding DMH neurons are glutamatergic. We also assessed the expression profile of neuropeptides and receptors and found high expression of CCK in tdTomato-positive neurons (Figure 6C). According to a previous study [26], glutamatergic neurons in the hypothalamus can be divided into 15 subtypes, and one of the markers is CCK. CCK-positive neurons express other markers (shown in Figure 6D). However, the gene profile of tdTomato-positive neurons did not match those of the subtypes listed in that study [26], indicating that either each sample contained neurons from multiple subtypes or that the labeled DMH neurons were an unidentified subtype.

**Figure 7:**
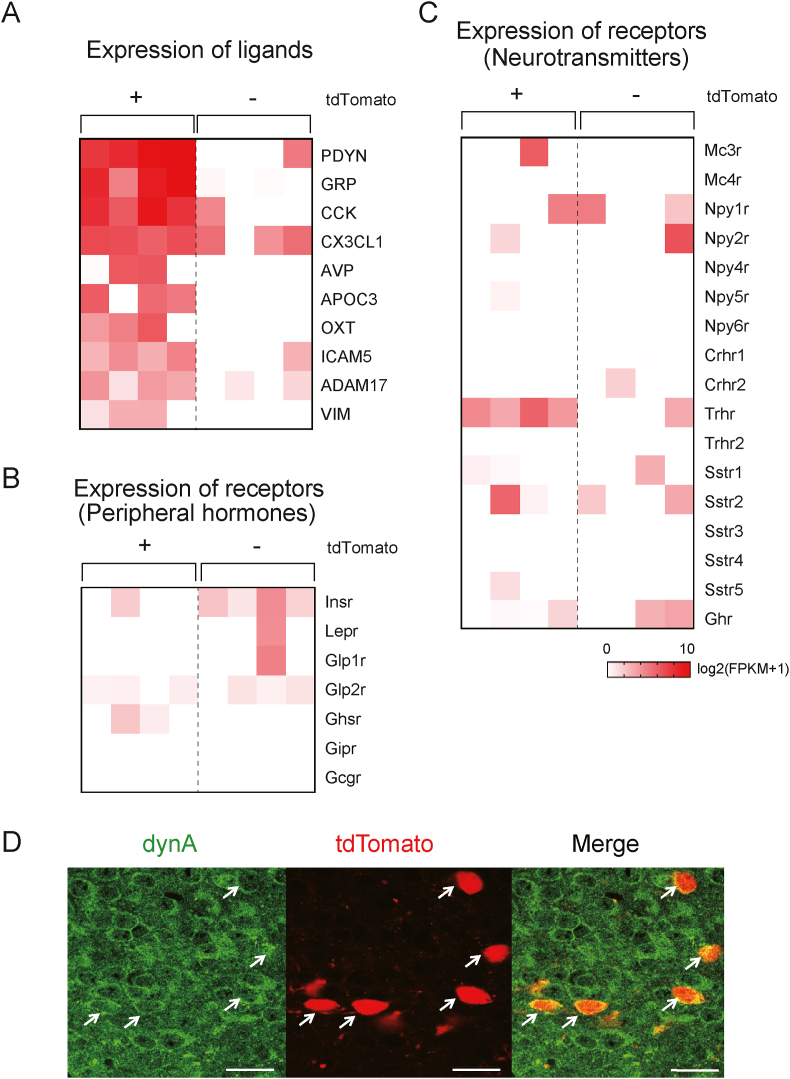
Refeeding-responsive DMH neurons express pdyn and CCK. A Heatmap of the gene expression of ligands and receptors involved in cell–cell communication. The gene list was obtained from a previous report [27]. Log_2_ (FPKM + 1). B, C Gene expression of receptors for peripheral hormones (B) and neurotransmitters (C) involved in metabolism. D Representative micrographs showing dynA (green) colocalization with TRAP-labeled tdTomato (red)-expressing neurons in the DMH. Scale bar: 20 μm.

To further investigate the unique transcriptional profile of tdTomato-positive neurons, we examined the gene expression of known ligands and receptors playing roles in cell–cell communication [27]. The gene expression heatmap for ligands were generated, and high expression of pdyn, gastrin-releasing peptide (GRP), and CCK were identified in tdTomato-positive neurons (Figure 7A). Pdyn is a precursor of dynorphins, the endogenous ligands for opioid receptors. Immunohistochemistry showed that 68.6 ± 9.6% (mean ± SEM, *n* = 3) of tdTomato-positive neurons colocalized with dynA (Figure 7D).

We also examined the expression of receptors for peripheral hormones and neurotransmitters (Figure 7B,C), and detected expression of the thyrotropin-releasing hormone receptor (Trhr) gene in tdTomato-positive neurons. The expression of receptors for peripheral metabolic hormones, including the insulin (Insr), leptin (Lepr) and glucagon-like peptide-1 (Glp1r) receptors, were not detected (Figure 7B). Collectively, our sequencing results suggest that refeeding-responsive DMH neurons labeled by the TRAP method are glutamatergic neurons expressing pdyn, GRP, CCK, and Trhr.

## 4 Discussion

Here, we identified a population of neurons in the compact part of the DMH activated by refeeding using the TRAP method. Chemogenetic activation and inhibition of TRAP-labeled neurons in the DMH decreased and increased food intake, respectively. The activation of the DMH neurons also promoted positive valence. The DMH neurons were identified as glutamatergic neurons and express the genes for pdyn, GRP, CCK, and Trhr. Our findings show that these DMH neurons have an important role in regulating food intake and are possibly involved in the satiety process for appetite and affectivity.

Homeostatic control of appetite is a complex process in which signals from peripheral hormones and the CNS are integrated to regulate feeding behaviors. Previous studies have shown that various subregions of the hypothalamus, including the ARC, VMH, PVH and lateral hypothalamus (LH), play important roles in the regulation of food intake. For example, POMC [5], NPY/AgRP [8,32], and dopamine neurons [33] in the ARC, steroidogenic factor 1 (SF1) neurons [34] in the VMH, corticotropin-releasing hormone neurons [21,35] in the PVH, and orexin neurons [36] in the LH have been reported to play roles in the regulation of feeding. Among these, POMC and NPY/AgRP neurons are considered first-order neurons that control feeding behavior, because genetic deletion of hormone receptors in these neurons significantly impacts feeding behavior and body weight. However, these neurons quickly respond to the onset of feeding or even the perception of food [37,38]. Therefore, it is unclear which neurons generate satiety.

The TRAP method allows one to examine neurons based on activity induced by refeeding. We used Arg3.1-CreER^T2^ because Arg3.1 has never been used in the study of the hypothalamus, and it could therefore identify novel cell populations. Compared with IHC for cFos, Arg3.1-IHC and TRAP-labeled neurons show different expression patterns in both DMH and ARC after refeeding in the present study. Thus, TRAP-labeled cells may be different from cFos-expressing cells. Although the activation of SF1 and POMC neurons can terminate feeding behavior [5,34], TRAP-labeled neurons in the VMH and ARC displayed a similar activation status in both fasted and re-fed states in our study. Some POMC and VMH neurons have been reported to express Arg3.1 in a model of inflammation [39], but POMC, SF1, and NPY/AgRP neurons may not be labeled by fasting and refeeding.

The gene expression profile of TRAP-labeled DMH neurons resembled that of CCK neurons reported in a previous study [26], however, our RNA sequencing analysis suggests their gene expression profile is nonetheless distinct from that of the reported subtypes. The secretion of the peripheral hormones insulin and GLP1 increases after food intake, and they modulate neural function to decrease hunger and food intake [40,41]. However, the expression of the receptors Insr, Lepr and Glp1r was not observed, suggesting that DMH neurons receive other afferent signals to regulate food intake (Figure 7). Trhr is the only receptor that was expressed specifically in the TRAP-labeled neurons. A subset of TRH neurons in the PVH is activated by refeeding [42], and injection of TRH into the third ventricle or medial hypothalamus, including the DMH, suppresses food intake after fasting [43]. TRH neurons may be involved in regulating the activity of TRAP-labeled neurons in the DMH.

Pdyn is a precursor of dynorphins, which are endogenous opioid peptides that signal via mu (MOP), kappa (KOP) and delta opioid (DOP) receptors. Dynorphins have a high affinity for KOP receptors, and activation of KOP receptors has an antinociceptive effect [44]. Dynorphins are also associated with negative valence, as KOP receptor activation in the nucleus accumbens (NAC) decreases dopamine release [45,46]. Previous studies suggest that the central opioid system is involved in the regulation of feeding behavior [47] and have highlighted the role of MOPs and other opioids, enkephalin and beta-endorphins, but not KOPs or dynorphins, in food intake and the rewarding effects of food [47,48].

Consistent with our findings, previous reports have implicated pdyn neurons in energy homeostasis. A study by Allison et al. identified neurons in the DMH expressing Lepr/pdyn using translating ribosome affinity purification and RNA sequencing analysis [49]. Ablation of Lepr in pdyn neurons alters energy expenditure in mice [49]. Garfield et al. demonstrated that Lepr/pdyn-expressing GABAergic DMH neurons project to AgRP neurons in the ARC and modulate feeding behavior [50]. However, in the current study, we did not detect Lepr expression or projections to the ARC, indicating that pdyn neurons labeled by our TRAP method are a different subpopulation of neuron. A study using ribosome phosphorylation identified pdyn-expressing neurons in the DMH that were activated during scheduled feeding [51]. Intracerebroventricular injection of a KOP receptor antagonist, norbinaltorphimine, increased food intake during scheduled feeding [51]. Taken together, these observations suggest that the DMH contains several subtypes of pdyn neurons that are involved in the regulation of feeding behavior and energy expenditure.

There are two forms of appetite, homeostatic and hedonic, and their regulatory mechanisms affect each other to maintain the balance between food intake and energy expenditure [52]. The experiencing of pleasure is a key factor in determining future behavioral actions [47]. Our findings indicate that activation of pdyn/CCK DMH neurons inhibits food intake and promotes positive valance, suggesting a key role of emotion in the transition from hunger (desire to eat) to satiety (termination of hunger). Numerous studies have reported on the rewarding effect of food and the role of mesolimbic pathways in producing the hedonic response [53]. Our findings suggest that pdyn/CCK neurons in the DMH play an important role in connecting the feeling of satisfaction and the termination of feeding behavior. To further elucidate the role of emotion, additional study of the neuronal networks upstream and downstream of the refeeding-responsive DMH neurons is needed.

This study has several limitations. First, the mice were singly housed the day before the measurement of food intake. Social isolation is known to modulate feeding behavior. CART fibers in the DMH are decreased by isolation stress [54] and an injection of CART into the DMH increases food intake [55]. However, rodents are kept in a single cage for at least one week in many types of research on social isolation [54,56,57]. The effect of overnight single cage housing on feeding behavior in our study is not clear. Second, although we found GFP-positive fibers in the PVH, SHi, and LS, it is not clear whether they are axon terminals or the ones just passing through. More experiments, such as optogenetic activation of axon terminals, are necessary to clarify the neuronal pathways from the DMH neuron. Third, although DMH neurons express Arg3.1 at 1 and 2 h after refeeding, our study did not measure the electrical responses of these neurons during refeeding. *In vivo* calcium imaging will help to understand the real–time activity of these neurons. Lastly, our RNA-sequencing data shows that the DMH neurons contain mRNA of POMC, AgRP, NPY, and Cartpt, which are well known to express in the ARC. NPY and Cart neurons are known to exist in the DMH [58], but POMC and AgRP are not. One possibility is that papain-mediated tissue digestion was not enough to separate fibers of POMC and AgRP neurons (which are densely found in the DMH [59]) from the TRAP-labeled DMH neurons. FPKM values of POMC and AgRP were 100 times less than that of CCK and these gene expressions were not constantly found in collected samples. While these data do not influence our conclusion, a better method of tissue digestion for the single-cell RNA sequence will be beneficial for studies of single-cell omics.

## 5 Conclusion

This study provides insight into the refeeding-responsive neurons in the hypothalamus under physiological conditions. We identified a novel subset of neurons in the DMH which is activated 1 h after refeeding to inhibit food intake. In addition, these DMH neurons can promote positive valence, which may affect feeding-related positive emotions. This neuronal circuit could be a potential therapeutic target for obesity and eating disorders.

## Author contributions

C.T. conceived this study and designed the experiments. D.I. performed most of the experiments, and C.T. supervised the entire study. I.Y., H.M. and S.X. performed the TRAP, GTT and ITT experiments. D.I. and T. Yonekura injected AAVs. S.X., T.I., S.Y. performed IHC. D.I., K. Kato, and S.X. conducted the CPP. I.Y., T. Yamasaki, T. Yonekura, K.O. and M.H. performed parts of the single-cell RNA sequence study. I.Y., D.I. and C.T. performed data analysis. I.Y., D.I., N.I., K. Kimura and C.T. wrote or contributed to the writing of the manuscript.

## References

[1] Heisler L.K., Lam D.D. (2017). An appetite for life: brain regulation of hunger and satiety. Current Opinion in Pharmacology.

[2] Blundell J. (1991). Pharmacological approaches to appetite suppression. Trends in Pharmacological Sciences.

[3] Morton G.J., Meek T.H., Schwartz M.W. (2014). Neurobiology of food intake in health and disease. Nature Reviews Neuroscience.

[4] Shimazu T., Minokoshi Y. (2017). Systemic glucoregulation by glucose-sensing neurons in the ventromedial hypothalamic nucleus (VMH). Journal of the Endocrine Society.

[5] Toda C., Santoro A., Kim J.D., Diano S. (2017). POMC neurons: from birth to death. Annual Review of Physiology.

[6] Toda C., Diano S. (2014). Mitochondrial UCP2 in the central regulation of metabolism. Best Practice & Research Clinical Endocrinology & Metabolism.

[7] Yanagi S., Sato T., Kangawa K., Nakazato M. (2018). The homeostatic force of ghrelin. Cell Metabolism.

[8] Aponte Y., Atasoy D., Sternson S.M. (2011). AGRP neurons are sufficient to orchestrate feeding behavior rapidly and without training. Nature Neuroscience.

[9] den Pol A.N., Acuna C., Davis J.N., Huang H., Zhang X. (2019). Defining the caudal hypothalamic arcuate nucleus with a focus on anorexic excitatory neurons. The Journal of Physiology.

[10] Krashes M.J., Shah B.P., Madara J.C., Olson D.P., Strochlic D.E., Garfield A.S. (2014). An excitatory paraventricular nucleus to AgRP neuron circuit that drives hunger. Nature.

[11] Jeong J.H., Lee D.K., Jo Y.-H. (2017). Cholinergic neurons in the dorsomedial hypothalamus regulate food intake. Molecular Metabolism.

[12] Yang L., Scott K.A., Hyun J., Tamashiro K.L., Tray N., Moran T.H. (2009). Role of dorsomedial hypothalamic neuropeptide Y in modulating food intake and energy balance. Journal of Neuroscience.

[13] Qian S., Chen H., Weingarth D., Trumbauer M.E., Novi D.E., Guan X. (2002). Neither agouti-related protein nor neuropeptide Y is critically required for the regulation of energy homeostasis in mice. Molecular and Cellular Biology.

[14] Deem J.D., Faber C.L., Morton G.J. (2021). AgRP neurons: regulators of feeding, energy expenditure, and behavior. FEBS Journal.

[15] Guenthner C.J., Miyamichi K., Yang H.H., Heller H.C., Luo L. (2013). Permanent genetic access to transiently active neurons via TRAP: targeted recombination in active populations. Neuron.

[16] Link W., Konietzko U., Kauselmann G., Krug M., Schwanke B., Frey U. (1995). Somatodendritic expression of an immediate early gene is regulated by synaptic activity. Proceedings of the National Academy of Sciences of the United States of America.

[17] Guillod-Maximin E., Lorsignol A., Alquier T., Penicaud L. (2004). Acute intracarotid glucose injection towards the brain induces specific c-fos activation in hypothalamic nuclei: involvement of astrocytes in cerebral glucose-sensing in rats. Journal of Neuroendocrinology.

[18] Schneider C.A., Rasband W.S., Eliceiri K.W. (2012). NIH Image to ImageJ: 25 years of image analysis. Nature Methods.

[19] Krashes M.J., Koda S., Ye C., Rogan S.C., Adams A.C., Cusher D.S. (2011). Rapid, reversible activation of AgRP neurons drives feeding behavior in mice. Journal of Clinical Investigation.

[20] Gomez J.L., Bonaventura J., Lesniak W., Mathews W.B., Sysa-Shah P., Rodriguez L.A. (2017). Chemogenetics revealed: DREADD occupancy and activation via converted clozapine. Science.

[21] Kim J., Lee S., Fang Y.-Y., Shin A., Park S., Hashikawa K. (2019). Rapid, biphasic CRF neuronal responses encode positive and negative valence. Nature Neuroscience.

[22] Bello-Arroyo E., Roque H., Marcos A., Orihuel J., Higuera-Matas A., Desco M. (2018). MouBeAT: a new and open toolbox for guided analysis of behavioral tests in mice. Frontiers in Behavioral Neuroscience.

[23] Hempel C.M., Sugino K., Nelson S.B. (2007). A manual method for the purification of fluorescently labeled neurons from the mammalian brain. Nature Protocols.

[24] Dobin A., Davis C.A., Schlesinger F., Drenkow J., Zaleski C., Jha S. (2013). STAR: ultrafast universal RNA-seq aligner. Bioinformatics.

[25] Liao Y., Smyth G.K., Shi W. (2014). FeatureCounts: an efficient general purpose program for assigning sequence reads to genomic features. Bioinformatics.

[26] Chen R., Wu X., Jiang L., Zhang Y. (2017). Single-cell RNA-Seq reveals hypothalamic cell diversity. Cell Reports.

[27] Ramilowski J.A., Goldberg T., Harshbarger J., Kloppmann E., Lizio M., Satagopam V.P. (2015). A draft network of ligand–receptor-mediated multicellular signalling in human. Nature Communications.

[28] Feetham C.H., O'Brien F., Barrett-Jolley R. (2018). Ion Channels in the paraventricular hypothalamic nucleus (PVN); emerging diversity and functional roles. Frontiers in Physiology.

[29] Johnson P.M., Kenny P.J. (2010). Dopamine D2 receptors in addiction-like reward dysfunction and compulsive eating in obese rats. Nature Neuroscience.

[30] Thanos P.K., Kim R., Cho J., Michaelides M., Anderson B.J., Primeaux S.D. (2010). Obesity-resistant S5B rats showed greater cocaine conditioned place preference than the obesity-prone OM rats. Physiology & Behavior.

[31] Batten S.R., Hicks K.B., Dwoskin L.P., Beckmann J.S. (2020). Toward isolating reward changes in diet-induced obesity: a demand analysis. Physiology & Behavior.

[32] Mercer R.E., Chee M.J.S., Colmers W.F. (2011). The role of NPY in hypothalamic mediated food intake. Frontiers in Neuroendocrinology.

[33] Zhang X., van den Pol A.N. (2016). Hypothalamic arcuate nucleus tyrosine hydroxylase neurons play orexigenic role in energy homeostasis. Nature Neuroscience.

[34] Choi Y.-H., Fujikawa T., Lee J., Reuter A., Kim K.W. (2013). Revisiting the ventral medial nucleus of the hypothalamus: the roles of SF-1 neurons in energy homeostasis. Frontiers in Neuroscience.

[35] Okamoto S., Kimura K., Saito M. (2001). Anorectic effect of leptin is mediated by hypothalamic corticotropin-releasing hormone, but not by urocortin, in rats. Neuroscience Letters.

[36] Diano S., Horvath B., Urbanski H.F., Sotonyi P., Horvath T.L. (2003). Fasting activates the nonhuman primate hypocretin (orexin) system and its postsynaptic targets. Endocrinology.

[37] Brandt C., Nolte H., Henschke S., Engström Ruud L., Awazawa M., Morgan D.A. (2018). Food perception primes hepatic ER homeostasis via melanocortin-dependent control of mTOR activation. Cell.

[38] Chen Y., Lin Y.-C., Kuo T.-W., Knight Z.A. (2015). Sensory detection of food rapidly modulates arcuate feeding circuits. Cell.

[39] Grinevich V., Kolleker A., Eliava M., Takada N., Takuma H., Fukazawa Y. (2009). Fluorescent Arc/Arg3.1 indicator mice: a versatile tool to study brain activity changes in vitro and in vivo. Journal of Neuroscience Methods.

[40] Krieger J.-P. (2020). Intestinal glucagon-like peptide-1 effects on food intake: physiological relevance and emerging mechanisms. Peptides.

[41] Woods S.C., Seeley R.J., Porte D., Schwartz M.W. (1998). Signals that regulate food intake and energy homeostasis. Science.

[42] Sánchez E., Singru P.S., Acharya R., Bodria M., Fekete C., Zavacki A.M. (2008). Differential effects of refeeding on melanocortin-responsive neurons in the hypothalamic paraventricular nucleus. Endocrinology.

[43] Suzuki T., Kohno H., Sakurada T., Tadano T., Kisara K. (1982). Intracranial injection of thyrotropin releasing hormone (TRH) suppresses starvation-induced feeding and drinking in rats. Pharmacology Biochemistry and Behavior.

[44] Schwarzer C. (2009). 30 years of dynorphins — new insights on their functions in neuropsychiatric diseases. Pharmacology & Therapeutics.

[45] Spanagel R., Herz A., Shippenberg T.S. (1990). The effects of opioid peptides on dopamine release in the nucleus accumbens: an in vivo microdialysis study. Journal of Neurochemistry.

[46] Valentino R.J., Volkow N.D. (2018). Untangling the complexity of opioid receptor function. Neuropsychopharmacology.

[47] Fulton S. (2010). Appetite and reward. Frontiers in Neuroendocrinology.

[48] Zhang M., Kelley A.E. (1997). Opiate agonists microinjected into the nucleus accumbens enhance sucrose drinking in rats. Psychopharmacology.

[49] Allison M.B., Patterson C.M., Krashes M.J., Lowell B.B., Myers M.G., Olson D.P. (2015). TRAP-seq defines markers for novel populations of hypothalamic and brainstem LepRb neurons. Molecular Metabolism.

[50] Garfield A.S., Shah B.P., Burgess C.R., Li M.M., Li C., Steger J.S. (2016). Dynamic GABAergic afferent modulation of AgRP neurons. Nature Neuroscience.

[51] Knight Z.A., Tan K., Birsoy K., Schmidt S., Garrison J.L., Wysocki R.W. (2012). Molecular profiling of activated neurons by phosphorylated ribosome capture. Cell.

[52] Andermann M.L., Lowell B.B. (2017). Toward a wiring diagram understanding of appetite control. Neuron.

[53] Volkow N.D., Wise R.A., Baler R. (2017). The dopamine motive system: implications for drug and food addiction. Nature Reviews Neuroscience.

[54] Nakhate K.T., Kokare D.M., Singru P.S., Subhedar N.K. (2011). Central regulation of feeding behavior during social isolation of rat: evidence for the role of endogenous CART system. International Journal of Obesity.

[55] Lau J., Herzog H. (2014). CART in the regulation of appetite and energy homeostasis. Frontiers in Neuroscience.

[56] Sun M., Choi E.Y., Magee D.J., Stets C.W., During M.J., Lin E.-J.D. (2014). Metabolic effects of social isolation in adult C57BL/6 mice. International Scholarly Research Notices.

[57] Izadi M., Radahmadi M., Ghasemi M., Rayatpour A. (2018). Effects of isolation and social subchronic stresses on food intake and levels of leptin, ghrelin, and glucose in male rats. Advanced Biomedical Research.

[58] Lee S.J., Verma S., Simonds S.E., Kirigiti M.A., Kievit P., Lindsley S.R. (2013). Leptin stimulates neuropeptide Y and cocaine amphetamine-regulated transcript coexpressing neuronal activity in the dorsomedial hypothalamus in diet-induced obese mice. Journal of Neuroscience.

[59] Wang D., He X., Zhao Z., Feng Q., Lin R., Sun Y. (2015). Whole-brain mapping of the direct inputs and axonal projections of POMC and AgRP neurons. Frontiers in Neuroanatomy.

